# Characteristics of vaginal microbiota in various cervical intraepithelial neoplasia: a cross-sectional study

**DOI:** 10.1186/s12967-023-04676-5

**Published:** 2023-11-16

**Authors:** Yiran Liu, Shuzhen Wang, Jun Liu, Mingrui Su, Xiaoli Diao, Xiaolong Liang, Jianxin Zhang, Qiuxi Wang, Yuxin Zhan

**Affiliations:** 1 Department of Obstetrics and Gynecology, Beijing Chaoyang Hospital Affiliated With Capital Medical University, 8 Gongti South Road, Chaoyang District, Beijing, 100020 China; 2 Department of Pathology, Beijing Chaoyang Hospital Affiliated With Capital Medical University, 8 Gongti South Road, Chaoyang District, Beijing, 100020 China

**Keywords:** Cervical Cancer (CC), Cervical Intraepithelial Neoplasia (CIN), Vaginal Microbiota (VMB), Human papillomavirus (HPV)

## Abstract

**Background:**

Precancerous lesions of cervical cancer exhibit characteristics indicative of natural progression. To prevent overtreatment of patients whose cervical intraepithelial neoplasia (CIN) in regression and to predict the onset of invasive cervical cancer at an early stage, we've identified the vaginal microbiome as a potential key factor, which is associated with both HPV infection and the various cervical intraepithelial neoplasia. This study aims to investigate the microbiome characteristics of patients with various cervical intraepithelial neoplasia.

**Methods:**

Utilizing high-throughput 16S ribosomal RNA (16S rRNA) sequencing technology, a description of the characteristics and community composition of Vaginal Microbiota (VMB) was conducted among 692 Chinese women infected with the High-risk Human Papillomavirus (HR-HPV).

**Results:**

As the grade of the lesions increased, the proportions of *Lactobacillus* and *Pseudomonas* demonstrated a significant declining trend, while the proportions of *Gardnerella*, *Dialister*, and *Prevotella* significantly increased. The diversity of the VMB was more significant in high-grade CIN. Furthermore, KEGG pathway enrichment analysis indicates that high-grade cervical intraepithelial neoplasia can inhibit various pathways, including those of phosphotransferase system, transcription factors, Fructose and mannose metabolism, amino sugar and nucleotide sugar metabolism, and galactose metabolism, which may contribute to the development of early cervical cancer symptoms.

**Conclusion:**

Patients with CIN exhibit a distinct vaginal microbial profile characterized by a decrease in *Lactobacillus* and *Pseudomonas*, and an increase in *Gardnerella*, *Prevotella*, and *Dialister*. The proliferation and diminution of these two types of microbial communities are interrelated, suggesting a mutual restraint and balance among them. Disruption of this regulatory balance could potentially lead to the onset of cervical lesions and carcinogenesis.

*Retrospectively registered:* This study was approved by the Ethics Committee of the Beijing Chaoyang Hospital affiliated with the Capital Medical University (NO.2023-S-415).

## Background

Cervical cancer (CC) is a malignant tumor originating from the cervix and ranks as the fourth most common type of gynecological malignancy globally [1], highlighting its grave impact on women's health [2]. CIN, closely related to invasive CC, encompasses cervical dysplasia and carcinoma in situ [3]. Depending on the extent of dysplastic cells in the cervical squamous epithelium, CIN can be divided into CIN1, CIN2, andCIN3 [4]. According to World Health Organization cervical pathological classification in 2014, CIN2 can be divided into two categories based on histological Cyclin-Dependent Kinase Inhibitor 2A (P16) results: low squamous intraepithelial lesions(LSIL) including CIN1 and P16-negative CIN2, and high squamous intraepithelial lesions(HSIL) including CIN3 and P16-positive CIN2 [5].

The occurrence of CC generally goes through CIN1, CIN2 and CIN3, some of them ultimately progressing into invasive carcinoma. During this process, CIN lesions may regress, persist, or progress in a diverse range of developments. However, it is worth noting that only a small proportion of cases progress to invasive carcinoma, thus necessitating increased attention towards patients with higher risk factors [6]. The forecast and surveillance of the natural progression of CIN with aim to reduce over-medicalization in patients who may have regressive lesions. Several studies have revealed notable differences in the types and proportions of VMB between healthy people and those with precancerous lesions or CC [7–9]. Such insights into the VMB present potential areas of exploration for future research.

Despite substantial evidence suggesting that certain HPV subtypes are major pathogens in the development and progression of CIN to CC [10–12], it has also been conclusively demonstrated that HPV infection alone is insufficient to cause CC [13–16]. Some studies have demonstrated a decline in the predominance of *Lactobacillus* and an increased prevalence of various harmful VMB in patients with precancerous lesions and CC. Notably, these alterations were found to correlate with elevated vaginal Potential of Hydrogen (PH) levels [17–19].

Recently, a potential interplay has been discovered among the composition of vaginal microbiome, HPV persistence, and CIN development. A microbiome characterized by increased levels of *G. vaginalis* and decreased levels of *L. iners*, *L. crispatus*, and *L. taiwanensis* may serve as a contributing factor [20]. Moreover, the VMB plays an indispensable role in the development of HPV-induced cervical tumors [18]. A healthy vaginal microbiota is typically dominated by *Lactobacillus* [7], which can produce lactic acid and create an acidic environment [21]. This helps to maintain vaginal health and preventing sexually transmitted infections [14, 15]. However, an imbalanced defense system may induce histological changes in the vaginal mucosa and cervical epithelium, thereby exerting selective pressure on the microbial community [8, 9, 22]. The most recent data suggest that bacterial vaginosis (BV), characterized by non-lactobacillus dominance (NLD), is associated with an elevated risk of HPV acquisition and reduced clearance. This condition could potentially lead to the development and progression of pre-cancerous lesions, thereby increasing the risk of CC [17–19, 23–27]. Although numerous studies have investigated the relationship between the VMB and CIN, it remains unclear which specific vaginal microenvironment promotes or inhibits disease progression and how to predict the natural disease progression through vaginal microbiota.

Our study aims to elucidate the compositional variations of VMB in different cervical lesions and identify key characteristic microbes associated with various grades of cervical abnormalities using a cross-sectional approach. The relationship between cervical carcinogenesis and high-risk HPV infection has been established [28]. However, the majority of HPV infections can naturally regress, with only a minority of persistent HPV infections leading to precancerous cervical lesions. Consequently, our primary task is to stratify the population with high-risk HPV infection, aiming to identify individuals who may develop precancerous cervical lesions or even undergo carcinogenesis at an early stage. Therefore, we will include patients with high-risk HPV infection but without cervical intraepithelial neoplasia as the control group in our study. Utilizing baseline data from an ongoing perspective study, we aim to establish a foundation for future research and concurrently conduct a prospective cohort study to reinforce the validation of our initial observational findings. By gaining a deeper understanding of the relationship between the VMB and cervical carcinogenesis, we will be able to adopt effective prevention and treatment strategies to prevent the occurrence of CC.

## Materials and methods

### Sample collection and study design

**Fig. 1:**
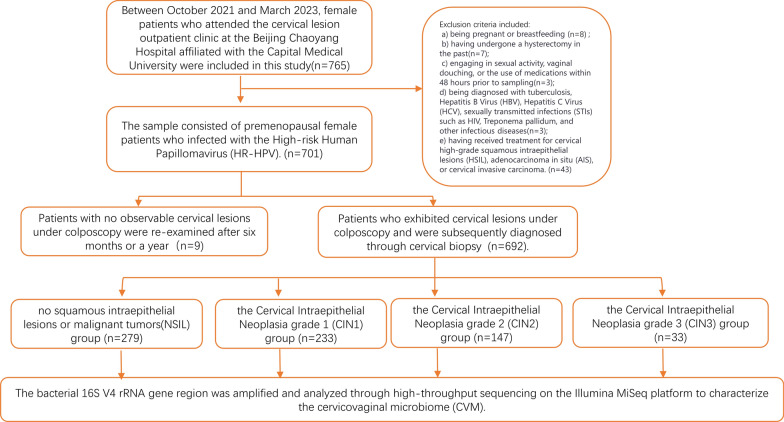
The flowchart illustrates the process of analyzing the cervical vaginal microbiota in patients who were initially filtered through inclusion and exclusion criteria, and subsequently divided into four groups via the method of cervical biopsy

Our Research design is shown in the following flowchart (Fig. 1). We designed a cross-Sectional study, during the period from October 2021 to March 2023. We specifically recruited premenopausal female patients who presented with HPV-positive results at outpatient clinic of Beijing Chaoyang Hospital, affiliated with the Capital Medical University. After applying the specified inclusion and exclusion criteria, we ultimately obtained a cohort of 692 patients. The inclusion criteria were as follows: (a) Women in their child-bearing years (b) HPV-positive results; (c) voluntary participation and the signing of a written informed consent form; and (d) willingness to cooperate during the collection of cervical and vaginal samples. The exclusion criteria were as follows: (a) being pregnant or breastfeeding; (b) having undergone a hysterectomy in the past; (c) being diagnosed with tuberculosis, Hepatitis B Virus (HBV), Hepatitis C Virus (HCV), sexually transmitted infections (STIs) such as Human Immunodeficiency Virus (HIV), Treponema pallidum, and other infectious diseases; and d) Long-term use of immunosuppressants, or having immune system diseases; (e) having received treatment for cervical high-grade squamous intraepithelial lesions (HSIL), adenocarcinoma in situ (AIS), or cervical invasive carcinoma.

Participants were interviewed to gather information related to factors associated with HPV infection. From biopsies performed under colposcopy, we classified 692 patients into four groups: NSIL group (n = 279), CIN1 group (n = 233), CIN2 group (n = 147), and CIN3 group (n = 33). In our study, the control group has consisted of patients who are infected with high-risk HPV but do not have cervical intraepithelial neoplasia. This study obtained approval from the institutional review board (NO.2023-S-415), and all patients provided written informed consent. All experiments were performed in accordance with relevant named guidelines and regulations.

Sterile cotton swabs were used to collect vaginal secretions from 692 women for vaginal microbiota examination. The cotton swab was swiftly immersed into a collection tube holding 1 ml of Phosphate-Buffered Saline(PBS). After briefly agitating the solution, the cotton swab was left in the tube, which was then promptly stored at − 80 °C in anticipation of Deoxyribonucleic Acid(DNA) extraction. Any sample collection needs to be excluded engaging in sexual activity, vaginal douching, or the use of medications within 48 h prior to sampling;

### Pathological biopsys

Women identified with cervical lesions through colposcopy underwent pathological biopsy. The pathology results and CIN classifications were then confirmed by two pathologists at Beijing Chaoyang Hospital.

### DNA extraction, PCR amplification and 16S rRNA sequencing

Genomic DNA from each sample was extracted following the standardized protocol using the Cetyltrimethylammonium Bromide(CTAB) method (CTAB, Nobleryder, China). The vaginal microbiome was assessed by sequencing the V4 region of the 16S rRNA, with the bacterial 16S primers F: GTGCCAGCMGCCGCGGTAA and R: GGACTACHVGGGTWTCTAAT. Amplification of PCR was performed using the Phusion® High-Fidelity Polymerase Chain Reaction(PCR) Master Mix with Guanine-Cytosine buffer(GC buffer) (New England Biolabs, USA). All PCR products were checked by gel electrophoresis. PCR products of 300–340 bp were purified using a universal DNA purification and recovery kit (TianGen, China, Catalog #: DP214). Libraries were constructed with the NEB (New England Biolabs, USA, Catalog #: E7430L) Next® Ultra™ II FS DNA PCR-free Library Prep Kit (NEB/E7430L). Upon satisfactory quantification via Qubit and Q-PCR, the libraries were sequenced on the NovaSeq 6000 (Illumina Novaseq6000, Illumina Inc., San Diego, CA, USA) using the PE 250 sequencing method.

### Sequence data filter, assemble and annotation

Unique barcodes were used to allocate paired-end reads to samples, followed by truncation by eliminating the barcode and primer sequence. The FLASH tool (V1.2.11, http://ccb.jhu.edu/software/FLASH/) [29], a rapid and accurate analysis tool, was employed to merge paired-end reads when some reads overlapped the read created from the opposite end of the identical DNA fragment. The resultant merged sequences were called raw tags. Quality filtering was performed on raw tags using the fastp software (Version 0.23.1) to achieve high-quality Clean Tags [30]. UCHIME Algorithm (http://www.drive5.com/usearch/manual/uchime_algo.html) was used to compare the tags with the reference database (Silva database (16S/18S), https://www.arb-silva.de/; Unite Database (ITS), https://unite.ut.ee/) to identify chimera sequences, which were then discarded[31]. This resulted in the final Effective Tags.

### Diversity classification

Operational taxonomic unit (OTU) clustering was conducted within the R 4.2.2 environment employing VSEARCH, which was subsequently followed by a diversity analysis utilizing the vegan 2.6–4 package. Taxonomic classifications were made based on taxonomic systems, and the corresponding alpha (α) and beta (β) diversities were calculated. The alpha diversity across four groups was demonstrated using observed OTU indices. Both weighted and unweighted principal component analyses (PCA) were executed to display beta diversity.

### Visualization software

Data visualization was performed using the ggplot2 3.4.1 package. We further investigated disease-associated bacterial taxa using Linear Discriminant Analysis Effect Size (LEfSe). Only bacterial taxa with an abundance exceeding 0.1% and Linear Discriminant Analysis (LDA) scores of ≤ − 3 or ≥ 3 were considered for further investigation. We employed the Greengenes 13.5 close reference OTU table for the prediction of the Kyoto Encyclopedia of Genes and Genomes(KEGG) level 3 functional table. Subsequent analyses of variances in the KEGG pathways across diverse sample groups were conducted using STAMP.

### Statistical analysis

Statistical analysis was performed using SPSS Statistics (version 20.0). Continuous variables are presented as mean ± standard deviation. Bioinformatic evaluations were conducted using the R package (Version 4.1). We calculated alpha diversity via the Shannon and Simpson indexes with the vegan package. For beta diversity, we employed principal-coordinate analysis (PCoA) using the Bray–Curtis algorithm. Categorical variables, represented as frequencies and percentages, were assessed through the chi-square test or Fisher’s exact test. Depending on data distribution, we used either the one-way analysis of variance (ANOVA) or the Kruskal–Wallis H test for multiple comparisons of continuous variables. Post hoc testing leveraged the Tukey–Kramer method, and a P value less than 0.05 was deemed statistically significant. In the STAMP software, Kruskal–Wallis tests were used for comparisons among multiple groups. For comparisons between two groups, we utilized the Welch's t-test. The Storey False Discovery Rate (FDR) method was employed for multiple test correction. The R software was utilized for the computation of diversity indices, and beta-diversity analysis was performed based on the Unweighted UniFrac distance.

## Result

### Characteristics of the Study Cohort

The subjects of this study were patients who visited the Cervical Lesion Outpatient Clinic at Beijing Chaoyang Hospital, affiliated with Capital Medical University, from October 2021 to March 2023. Table 1 presents the demographic data of the study population. There were no statistically significant differences among groups in terms of age (P = 0.202), Gravidity (P = 0.190), Parity (P = 0.300), Body Mass Index (BMI) (P = 0.614), age at first sexual intercourse (P = 0.810), sex age (P = 0.333), number of sexual partners (P = 0.066), native place (P = 0.890), family history of cancer (P = 0.801), history of HPV infection (P = 0.794), contraceptive methods (P = 0.750), HPV vaccine(P = 0.426)or smoking (P = 0.778) (P > 0.05 for all). From the figure, it can be observed that there are statistically significant differences in the TCT results and colposcopy outcomes among the four groups (P < 0.05).

**Table 1:** Description of study population

Characteristic	Data for:				P value
	NSIL group (n = 279)	CIN1 group (n = 233)	CIN2 group (n = 147)	CIN3 group (n = 33)	
Age (years)	35.49 ± 7.00	34.24 ± 7.53	34.88 ± 6.46	35.94 ± 6.86	0.202
Gravidity	1.45 ± 1.56	1.18 ± 1.31	1.39 ± 1.42	1.48 ± 1.46	0.190
Parity	0.58 ± 0.71	0.49 ± 0.63	0.63 ± 0.72	0.58 ± 0.75	0.300
Body mass index(kg/m^2^)	21.45 ± 3.35	21.49 ± 3.23	21.14 ± 3.01	21.85 ± 3.64	0.614
Age of first sexual(years) intercourse	21.77 ± 3.23	21.63 ± 3.11	21.69 ± 3.17	22.18 ± 3.31	0.810
Sexual age(years)	13.72 ± 6.85	12.61 ± 7.20	13.19 ± 6.82	13.76 ± 6.58	0.333
Number of sexual partners	2.92 ± 2.45	3.06 ± 2.32	2.96 ± 1.87	3.36 ± 2.64	0.710
Richness index^a^	283.16 ± 214.11	301.13 ± 217.65	356.93 ± 280.61	282.15 ± 239.79	0.017
CHAO1 index^a^	427.92 ± 301.70	465.17 ± 309.90	518.29 ± 372.63	429.60 ± 332.13	0.048
ACE index^a^	449.03 ± 308.44	488.70 ± 320.63	540.30 ± 378.92	447.06 ± 343.05	0.051
Shannon index^a^	1.32 ± 0.62	1.40 ± 0.59	1.57 ± 0.76	1.77 ± 0.84	< 0.001
Simpson index^a^	0.47 ± 0.20	0.50 ± 0.20	0.54 ± 0.21	0.61 ± 0.21	< 0.001
Invsimpson index^a^	2.46 ± 2.26	2.65 ± 1.81	3.20 ± 3.22	4.47 ± 5.75	< 0.001
Native place					0.890
Beijing	72 (25.81%)	62 (26.61%)	34 (23.13%)	8 (24.24%)	
Others	207 (74.19%)	171 (73.39%)	113 (76.87%)	25 (75.76%)	
Education					0.012
Junior high school and below	21 (7.55%)	21 (9.01%)	19 (12.93%)	6 (18.18%)	
Junior high school to high school	59 (21.22%)	45 (19.31%)	45 (30.61%)	4 (12.12%)	
Bachelor degree or above	198 (71.22%)	167 (71.67%)	83 (56.46%)	23 (69.70%)	
Family history of cancer					0.801
No	189 (67.74%)	151 (64.81%)	94 (63.95%)	23 (69.70%)	
Yes	90 (32.26%)	82 (35.19%)	53 (36.05%)	10 (30.30%)	
History of HPV infection					0.794
No	162 (58.06%)	127 (54.51%)	79 (53.74%)	19 (57.58%)	
Yes	117 (41.94%)	106 (45.49%)	68 (46.26%)	14 (42.42%)	
Contraceptive methods					0.750
Condom	206 (73.84%)	176 (75.54%)	109 (74.15%)	22 (66.67%)	
Other methods or no contraception	73 (26.16%)	57 (24.46%)	38 (25.85%)	11 (33.33%)	
HPV vaccine					0.426
Unvaccinated	200 (71.68%)	168 (72.10%)	109 (74.15%)	28 (84.85%)	
Vaccinated	79 (28.32%)	65 (27.90%)	38 (25.85%)	5 (15.15%)	
Smoking					0.778
Non-smoking	252 (90.32%)	211 (90.56%)	132 (89.80%)	28 (84.85%)	
Smoking	27 (9.68%)	22 (9.44%)	15 (10.20%)	5 (15.15%)	
TCT					< 0.001
NSIL	212 (76.26%)	129 (55.36%)	76 (51.70%)	14 (42.42%)	
ASCUS	42 (15.11%)	55 (23.61%)	35 (23.81%)	5 (15.15%)	
LSIL	22 (7.91%)	46 (19.74%)	27 (18.37%)	7 (21.21%)	
ASC-H	2 (0.72%)	2 (0.86%)	4 (2.72%)	3 (9.09%)	
HSIL	0 (0.00%)	1 (0.43%)	5 (3.40%)	4 (12.12%)	
Transformation zone (TZ)					0.064
TZ-1	206 (73.84%)	180 (77.25%)	125 (85.03%)	27 (81.82%)	
TZ-2	36 (12.90%)	31 (13.30%)	17 (11.56%)	3 (9.09%)	
TZ-3	37 (13.26%)	22 (9.44%)	5 (3.40%)	3 (9.09%)	
Colposcopic impression					< 0.001
No signs of abnormalities	57 (20.43%)	45 (19.31%)	23 (15.65%)	3 (9.09%)	
LSIL	199 (71.33%)	152 (65.24%)	84 (57.14%)	4 (12.12%)	
HSIL	23 (8.24%)	36 (15.45%)	40 (27.21%)	26 (78.79%)	

N number of available values, *SD* standard deviation

*VMB* Vaginal Microbiota, *CHAO1* Chao1 Richness Estimator, *ACE* Abundance-based Coverage Estimator, *HPV* Human Papillomavirus, *TCT* Thinprep Cytologic Test, *NSIL* Negative for Squamous Intraepithelial Lesion, *ASCUS* Atypical Squamous Cells of Undetermined Significance, *LSIL* Low-grade Squamous Intraepithelial Lesion, *ASC-H* Atypical Squamous Cells cannot exclude HSIL, *HSIL* High-grade Squamous Intraepithelial Lesion, *TZ* Transformation Zone

Results are presented as Mean ± SD for continuous variables and N(%) for categorical variables

*P-values were determined using the Kruskal–Wallis test for continuous variables, Fisher's exact test for categorical variables with expected counts < 10, and the Chi-squared test for counts ≥ 10

### Composition and relative abundance of the vaginal microbiota

**Fig. 2:**
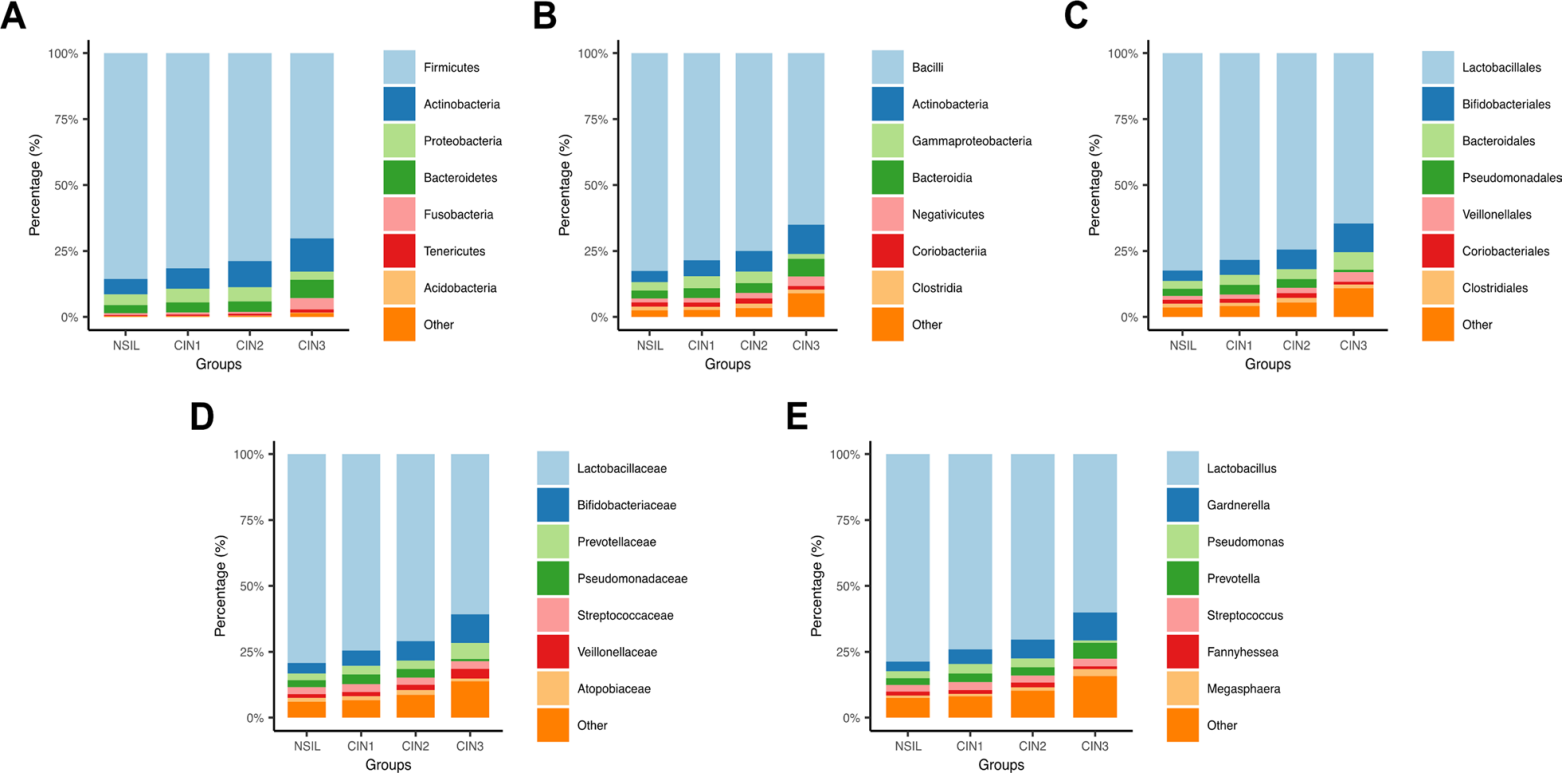
Composition classification and abundance comparison of vaginal microbiota. Comparison of vaginal microbiota at the levels of phylum (A), class (B), order (C), family (D) and genus (E) in the NSIL group, CIN I group, CIN II group and CIN III group

This study conducted a microbial genomics analysis (using16S rRNA gene sequencing) on vaginal discharge samples collected from four groups of patients, with a total of 692 samples. There were significant differences in the relative abundance of microbial bacteria among varying degrees of cervical lesions. At the phylum level, the main vaginal microbial communities among the four groups of patients were Firmicutes, Actinobacteria, Proteobacteria, Bacteroidetes, and Fusobacteria. Notably, the relative abundance of Actinobacteria, Bacteroidetes, and Fusobacteria was significantly higher in the CIN3 group than in other groups, while Firmicutes was significantly lower (Fig. 2A).

At the class level, the core vaginal microbial communities among patients consisted of Bacilli, Actinobacteria, Bacteroidia, Gammaproteobacteria, Negativicutes, Coriobacteriia and Clostridia. As the severity of cervical lesions increases, Bacilli and Gammaproteobacteria gradually decrease, while Actinobacteria, Bacteroidia, and Negativicutes show a significant increase (Fig. 2B).

At the order level, Lactobacillales, Bifidobacteriales, Bacteroidales, and Pseudomonadales were found to be the main microbial communities among the vaginal microbes in the four groups. Additionally, a lower relative abundance of Lactobacillales was observed in the CIN3 group while the abundance of Bacteroidales was higher (Fig. 2C).

The core microbial communities in the four groups at the family level were mainly composed of Lactobacillaceae, Bifidobacteriaceae, Prevotellaceae, Pseudomonadaceae, and Streptococcaceae (Fig. 2D).

Similarly, at the genus level, *Lactobacillus*, *Gardnerella*, *Prevotella*, *Pseudomonas*, and *Streptococcus* were the main bacteria in the patients' vaginal microbiome (Fig. 2E). The concentrations of both the genus *Lactobacillus* and the genus *Pseudomonas* exhibit a continuous declining trend, with the CIN3 group demonstrating the lowest levels of these two genera. (Fig. 2F). In contrast, the levels of *Gardnerella* and *Prevotella* increased. These findings suggest that the progression of CIN may be related to the relative abundance ratios of dominant microbial communities.

### Diversity analysis of α

**Fig. 3:**
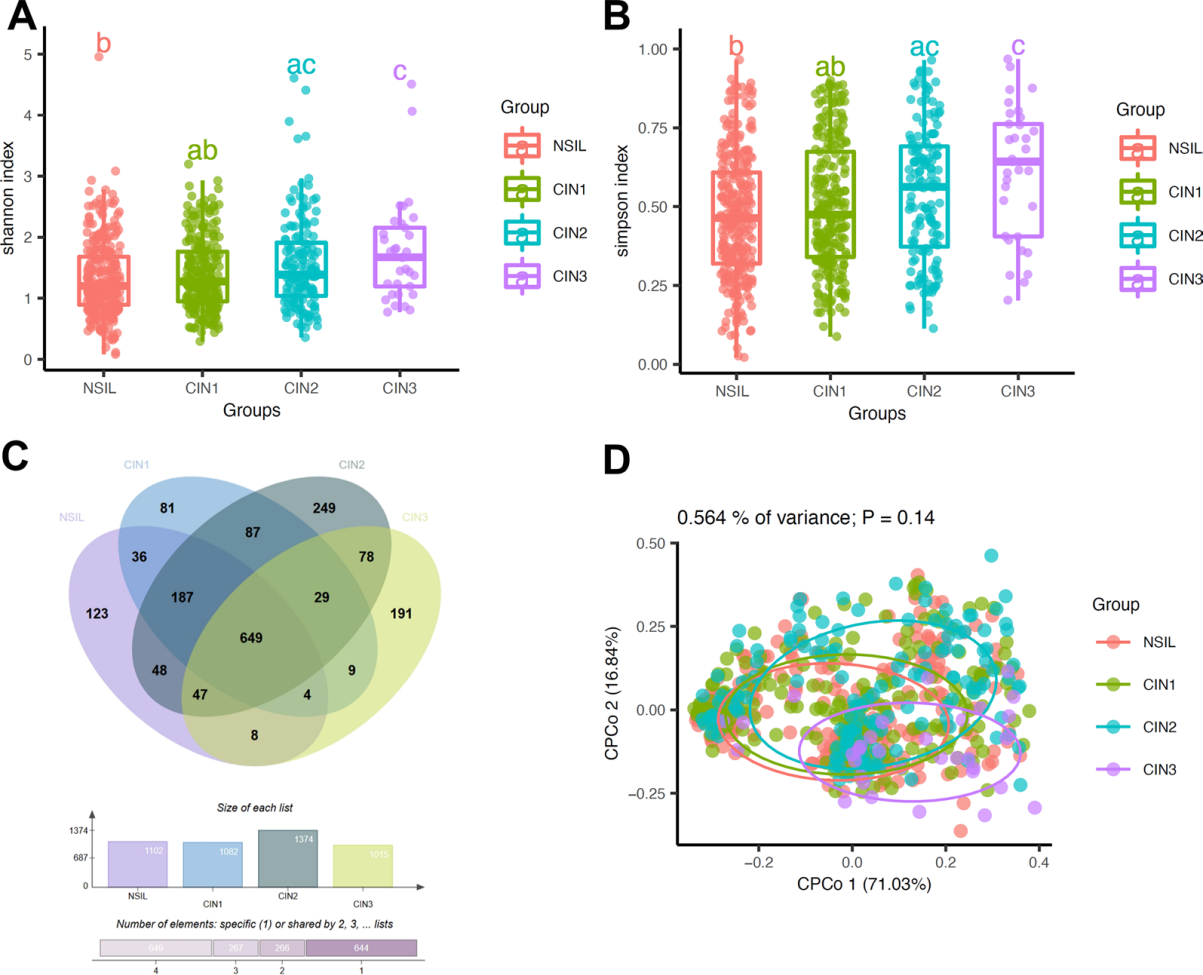
The alpha and beta diversity between the NSIL, CIN1, CIN2 and CIN3 groups. **A** Shannon index among four groups. **B** Simpson index among the four groups. **C** Total species among the four groups. **D** Principal-coordinate analysis among the four groups. A letter annotation system is employed to depict statistical relationships between data groups, utilizing letters "A," "B," "AB," "AC," and "C". Unique letters indicate statistically significant differences (P < 0.05), while letter combinations show no significant difference. Specifically, "B" different from "C" and "AC", but not from "AB". "AB" and "AC" are statistically similar, as are "AC" and "C", but "AB" different from "C"

The alpha diversity in each group was evaluated using Simpson and Shannon indices according to the species level. As shown in Fig. 3A, we observed significantly high values of the mean Shannon indexes in the patients of the CIN3 groups compared with those of the NSIL group (P < 0.05). Moreover, we also observed significantly low Chao1 indexes in the patients of NSIL group compared with those of the CIN1, CIN2 and CIN3 group(P < 0.05), whereas no significant difference was found between the patients of the CIN1 and CIN2 group. In summary, the CIN3 group harbored a greater variety of vaginal microbial communities compared to the other groups.

### β Diversity analysis

As the cervical lesions progressed, there was no significant increase in the total number of microbial species across the groups. Specifically, the NSIL group had 1,102 species, the CIN1 group had 1,082 species, the CIN2 group had 1,374 species, and the CIN3 group reported 1,015 species (see Fig. 3C). The data doesn't indicate a direct correlation between the progression of cervical lesions and the diversity of the cervical microbiome. Furthermore, the principal coordinate analysis (PCoA) utilizing the Bray–Curtis statistical method, as illustrated in Fig. 3D, revealed no significant differences in beta diversity among the four groups(P = 0.14).

### Comparison of differences in vaginal microbial community by LEfSe analysis and stamp

**Fig. 4:**
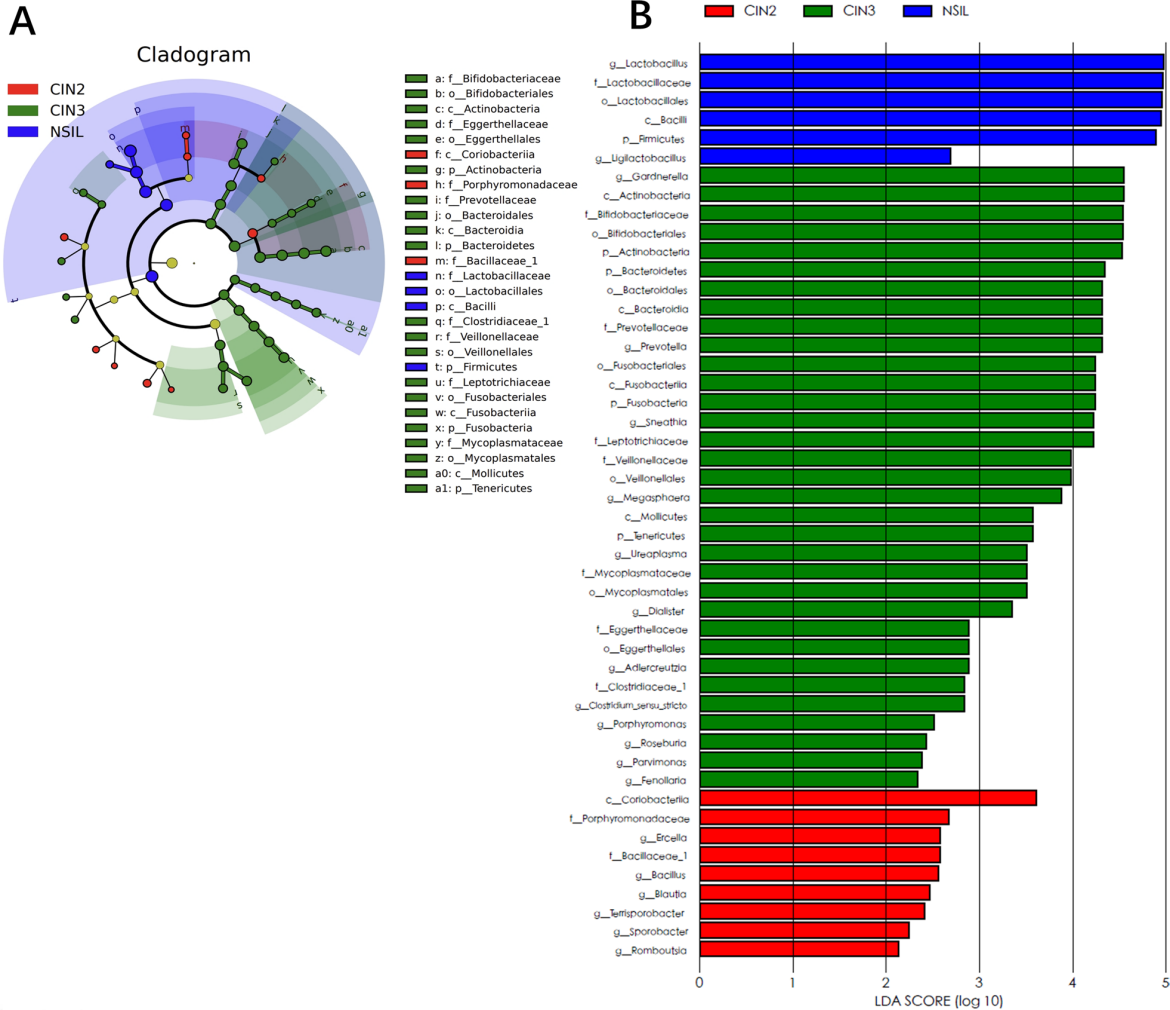
The LDA effect size analysis (LEfSe) results highlights distinctions in the cervical microbiome based on cervical lesion classification. **A** The cladogram depicts differentially abundant microbial clades and nodes. The circles radiating from the inside out represent the taxonomic level from phylum to genus. Specific taxonomies at each level are represented by individual circles, with their diameter being indicative of their relative abundance. **B** Microbial taxa with a significant differential abundance and LDA score > 2 are illustrated in the histogram. Bar lengths convey the effect sizes that notably differentiate the NSIL, CIN1, CIN2, and CIN3 groups

The Linear Discriminant Analysis Effect Size (LEfSe) was employed to distinguish differences in bacterial abundance that might be linked to the progression of cervical lesions (Fig. 4). Furthermore, taxonomic units with LDA scores exceeding the threshold (LDA > 2) on the LDA score graph are considered as significant biomarkers for each group. From the clustering diagram, it is clear that taxonomic units at all levels (from phylum to genus) significantly differ among the four groups. We discovered that *Lactobacillus* and *Ligilactobacillus* genus were considered a potential biomarker for distinguishing HPV infection from other cervical lesions. Moreover, the *Ercella, Bacillus, BLautia, Terrisporobacter, Sporobacter, Romboutsia* genus was identified as a prominent microbiota indicator for patients with CIN2. Furthermore, the genera *Prevotella*, *Gardnerella*, *Sneathia, Megasphaera, Dialister and Ureaplasma* were recognized as significant microbial indicators for the CIN3 group. In conclusion, the LEfSe analysis reveals distinctive microbial features in each group, indicating the existence of unique microbial community structures.

To further investigate the specific microbial differences in various stages of cervical cancer, we used STAMP to analyze the taxonomic profiles of the four groups. To better assess the influence of the abundance of each component (species) on the variation in flora, we conducted a linear discriminant analysis (LDA) utilizing a non-parametric Kruskal-Walli’s rank sum test.

**Fig. 5:**
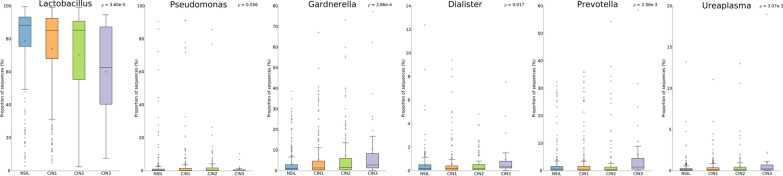
Statistical evaluation of variations in microbial genera across distinct cervical scraping sample groups

**Fig. 6:**
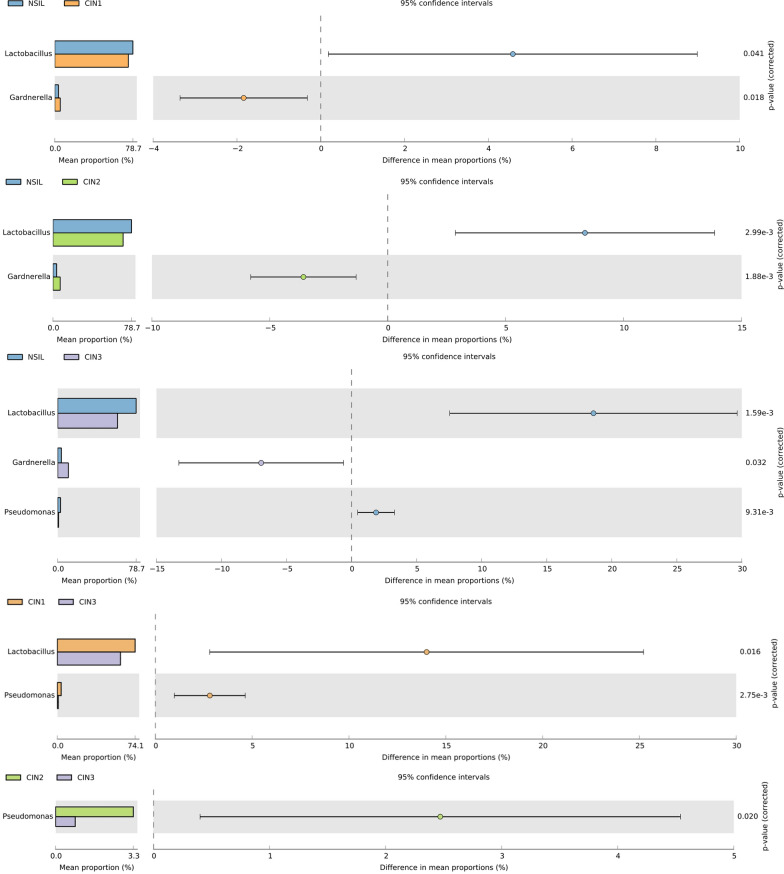
Statistical assessment of variations in microbial genera among distinct cervical scraping sample groups. Differential genera were identified based on discrepancies in mean proportions within 95% confidence intervals

As illustrated in Fig. 5, with the escalation of lesion grades, there's a discernible decline in the relative abundance of *Lactobacillus*, reaching a statistically significant difference with P values(corrected)3.60e-5. It can also be observed that *Pseudomonas* significantly decreases in CIN3 lesions, but there is no significant statistical difference among the four groups. Conversely, the relative abundance of *Gardnerella*, *Dialister*, *Prevotella*, and *Ureaplasma* consistently increased. This data underscores the pivotal roles of *Gardnerella*, *Dialister*, *Prevotella*, and *Ureaplasma* in high-grade cervical intraepithelial neoplasia. Additionally, based on significant variations among the groups, *Lactobacillus*, *Gardnerella*, and *Pseudomonas* showed the greatest difference in mean proportions with 95% confidence intervals. In the NSIL group, the abundance of *Lactobacillus* was notably higher than in other CIN groups, displaying a statistical significance (Fig. 6). As lesion grades progressed, the abundance of *Lactobacillus* continuously diminished. While there was a clear distinction between the CIN1 and CIN3 groups, differences between adjacent lesion grades were not statistically significant. For *Gardnerella*, significant statistical differences were observed between the NSIL group and other CIN groups. Although there was an upward trend in the abundance of this bacterium across CIN1, 2, and 3 groups with the progression of lesion grades, the differences among these three groups were not statistically significant.

In the CIN3 group, *Pseudomonas* demonstrated a pronounced reduction, exhibiting significant statistical differences when compared to the other three groups.

### KEGG pathway analysis of differential florae for

#### biomarker prediction

**Fig. 7:**
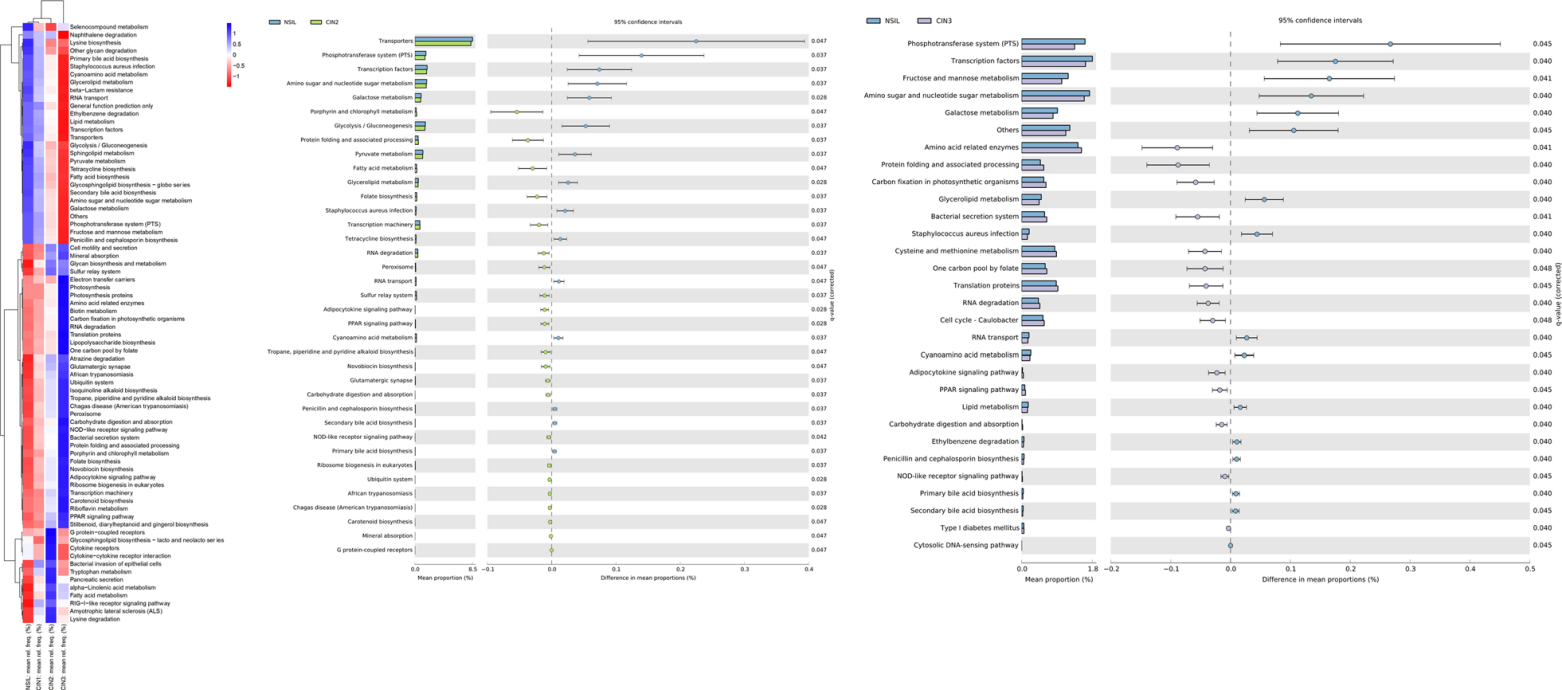
Heatmap representation of enriched KEGG pathways across the four cervical scraping sample groups. Functional gene composition of the metagenome was predicted using PICRUSt, leveraging the Greengenes 13.5 close reference OTU table to anticipate the KEGG level 3 functional table. Variations in the KEGG pathways among sample groups were assessed using STAMP

Utilizing the Phylogenetic Investigation of Communities by Reconstruction of Unobserved States (PICRUSt) software, we predicted the metagenome's functional gene composition from the OTU data. This prediction was based on the inference of gene function profiles from the 16S rRNA full-length sequences of the examined bacterial genomes. These profiles were sourced from the comprehensive range of archaeal and bacterial domains present in the Greengenes database. The composition of the sequenced bacterial groups was integrated into this database to anticipate the metabolic functions of the bacterial assemblies. A heatmap generated from the KEGG pathway analysis revealed distinct gene functions across the four groups (as illustrated in Fig. 7).

When comparing the NSIL group to the CIN2 group, we observed a decline in several metabolic pathways in the CIN2 group. This includes Transporters, Phosphotransferase system (PTS), Transcription factors, Amino sugar and nucleotide sugar metabolism, Galactose metabolism, Glycolysis, and Pyruvate metabolism. Conversely, the metabolic activities of Porphyrin and chlorophyll metabolism, Protein folding and associated processing, and Fatty acid metabolism were enhanced in the CIN2 group. In contrast to the NSIL group, the CIN3 group displayed a decreased metabolic activity in PTS, Transcription factors, Fructose and mannose metabolism, Amino sugar and nucleotide sugar metabolism, and Galactose metabolism. However, there was an increase in the metabolic activities of Amino acid related enzymes, Protein folding and associated processing, Carbon fixation in photosynthetic organisms, and Bacterial secretion system. This trend suggests that as the grade of cervical intraepithelial neoplasia escalates, certain biological processes are suppressed, while others are promoted. Notably, higher-grade lesions exhibit similar metabolic patterns. Additionally, we analyzed other metabolic pathways in heatmap, further delineating the differences among the groups.

## Discussion

Despite substantial evidence suggesting that certain HPV subtypes are major pathogens in the development and progression of CIN to CC, it has also been conclusively demonstrated that merely having HPV infection is insufficient to cause CC [13–16] It is postulated that particular vaginal microbiota might influence the onset of CIN through certain pathways. But by what mechanism does the VMB dominate the onset and severity of CIN? There are no predictable experimental results.

Preliminary studies suggest that a VMB dominated by anaerobic or facultative *Lactobacillus* bacteria, combined with a deficiency in lactic acid bacteria, can trigger an increase in the ratio of L-lactic acid to D-lactic acid produced by vaginal epithelial cells. This shift can elevate the expression of extracellular matrix metalloproteinase inducers and the activity of metalloproteinase-8. The latter can compromise the integrity of the cervical epithelium, facilitating HPV's transition, anchoring, replication, and spread in the cervical region, ultimately leading to CIN. Furthermore, dominant *Lactobacillus* Community State Types (CST I, CST II, and CST III) can maintain a healthy vaginal environment by producing antimicrobial agents, competitively inhibiting pathogenic bacterial and viral adhesion, and maintaining a low pH and localized anti-inflammatory microenvironment [32]. This is vital in mitigating the onset of high-grade CIN [33, 34].

In reproductive-aged females, the vaginal microbiota predominantly comprises *Lactobacillus*, accounting for approximately 80%, which sustains a pH range of 3.8 to 4.5 [35] Prior investigations have elucidated a correlation between decreased levels of cervical vaginal *Lactobacillus* and an increased susceptibility to HPV infection and the emergence of precancerous cervical lesions [32].

In our study, we observed a consistent decline in the proportions of both *Lactobacillus* and *Pseudomonas* genera as the severity of CIN increased, with the most significant reduction observed in the CIN3 group. The relationship between *Pseudomonas* and the severity of CIN has been identified. However, the specific role of *Pseudomonas* in HPV infection or its regression remains to be elucidated in subsequent studies.

Our findings align with previous research, confirming an increase in species diversity within the vaginal microenvironment as cervical lesions progress. Utilizing amplicon sequencing and subsequent analyses, we explored the potential links between cervical lesions at different stages and the vaginal-cervical microbiota, identifying characteristic microbial signatures associated with high-grade CIN. Additionally, we noted a gradual increase in the proportions of specific genera such as *Gardnerella*, *Dialister*, and *Prevotella* as precancerous cervical lesions developed, with Community State Type IV (CST IV), characterized by a dominance of these bacteria, showing increased susceptibility.

This indicates a mutual balance between the microbial communities consisting of *Lactobacillus* and *Pseudomonas* and those consisting of *Gardnerella*, *Dialister*, and *Prevotella*. Disruption of this balance could potentially lead to the onset of precancerous cervical lesions. Restoration of the vaginal microbiota balance may signify a reversal in the disease progression.

However, it is also noted that there is some controversy regarding whether the growth and decline of these two microbial communities determine the direction of lesion development. Furthermore, the specific roles of the three genera considered "harmful"—*Gardnerella*, *Dialister*, and *Prevotella*—in disease outcome are not yet fully understood, despite their seemingly crucial impact in smaller quantities.

*Lactobacillus iners* is a dominant bacterium in the vaginal microbiome. Studies have indicated that when there is an imbalance in the vaginal microecology, leading to a reduction in *Lactobacillus* levels, the infection rate of HR-HPV is significantly higher compared to women dominated by *Lactobacillus* [36]. This suggests that a decrease in *Lactobacillus* might promote the occurrence of HR-HPV infections. Further research has also shown that women who use *Lactobacillus* rhamnosus BMX 54 long-term, especially those already infected with HPV and accompanied by bacterial vaginosis or vaginitis, can mitigate viral infections by restoring the ecological balance of the vagina [37]. This offers an interesting perspective that reshaping the vaginal environment to be dominated by *Lactobacillus* could effectively reduce the rate of HPV infections.

In conclusion, while our study sheds light on possible connections and presents intriguing phenomena, further research is warranted to gain a clearer understanding of the precise relationships between these microbial communities and the development of cervical lesions.

Diversity analysis indicates an increase in the complexity of VMB with disease progression. Both species diversity and richness in the CIN3 group were higher than those in the other groups, suggesting a potential correlation between increased microbial diversity and the severity of cervical lesions. Therefore, an increase in VMB diversity throughout disease progression may serve as a potential biomarker for disease progression.

Beyond identifying shifts in the taxonomic structure during cervical cancer development, we also evaluated the functional attributes of the microbiome using KEGG pathway enrichment analysis [38]. With the onset of high-grade CIN, metabolic activities of amino acid-related enzymes, protein folding and associated processing, and bacterial secretion systems were significantly amplified. These metabolic shifts can elucidate the accelerated progression observed during high-grade CIN onset. Additionally, key sugar metabolic processes, including glycolysis/gluconeogenesis, fructose and mannose metabolism, galactose metabolism, and pyruvate metabolism, play pivotal roles in providing energy substrates for bacterial microbiota.

Given the frequent fluctuations in the vaginal microbiota, which are influenced not only by the severity of cervical lesions but also by various external factors, our study selectively included premenopausal women, ensuring no significant age differences among the groups. To enhance the reliability and accuracy of our findings, we also accounted for and balanced several confounding variables across the groups, including gravidity and parity, BMI, age at first sexual intercourse, age at initiation of sexual activity, number of sexual partners, place of origin, educational level, family history of cancer, history of HPV infection, contraceptive methods, HPV vaccination status, and smoking habits.

We conducted a thorough study using amplicon sequencing to explore the possible links between different stages of cervical lesions and the vaginal-cervical microbiota. However, our study has some limitations. First, it was carried out in a single center without a separate validation group, highlighting the need for broader, multi-center studies to confirm our findings. Additionally, our grasp on the vaginal microbiota is still basic. Considering the inherent variability of the vaginal microbiota and its sensitivity to numerous external influences, we made a concerted effort to account for a wide range of potential variables, ensuring a balanced representation across groups through meticulous selection. Nonetheless, there may still be additional factors, such as variations in HPV infection types, that have not been accounted for in our study. Given the inherent limitations of our cross-sectional study, we plan to continue monitoring this group of patients for subsequent cohort studies to validate our current findings. In summary, we've pointed out possible connections between cervical lesion stages and microbiota, but more research is needed to understand the cause and underlying processes.

Currently, the amalgamation of HPV DNA testing with ThinPrep Cytologic Test (TCT), colposcopy, and biopsy remains the clinical gold standard for CIN and CC screening and diagnosis. Recently, non-invasive gut microbiome models have been established to diagnose and predict several cancer types, including colorectal, breast, and liver cancers [39]. Drawing parallels, we hypothesize that the composition of the vaginal microbiome could be a promising biomarker for predicting CIN in patients. Echoing prior research, we postulate that the *Lactobacillus* genus could be a robust biomarker for predicting HPV infections. Pathogenic anaerobic bacteria, such as *Gardnerella*, *Prevotella*, and *Dialister*, might serve as potential biomarkers for predicting cervical lesions. In the future, it may be possible to expedite HPV virus clearance and delay or even reverse the progression of pre-cancerous cervical lesions by manipulating the distribution of microbial communities in the vaginal environment through biologics.

## Conclusion

In conclusion, the vaginal microbiota plays a significant role in the progression of cervical intraepithelial neoplasia (CIN) to cervical cancer (CC). This study provides new insights for the future diagnosis and treatment of cervical cancer.

## Data Availability

The datasets used and/or analysed during the current study are available from the corresponding author on reasonable request.

## Abbreviations

CIN: Cervical intraepithelial neoplasia
16S rRNA: 16S Ribosomal RNA
HR-HPV: High-risk Human Papillomavirus
CC: Cervical cancer
VMB: Vaginal microbiota
HPV: Human Papillomavirus
LSIL: Low squamous intraepithelial lesions
HSIL: High squamous intraepithelial lesions
BV: Bacterial vaginosis
NLD: Non-lactobacillus dominance
HBV: Hepatitis B virus
HCV: Hepatitis C virus
STIs: Sexually transmitted infections
AIS: Adenocarcinoma in situ
OUT: Operational taxonomic unit
PCA: Principal component analyses
LEfSe: Linear discriminant analysis effect size
LDA: Linear discriminant analysis
KEGG: Kyoto Encyclopedia of Genes and Genomes
PCoA: Principal-coordinate analysis
ANOVA: Analysis of variance
FDR: False discovery rate
BMI: Body mass index
PICRUSt: Phylogenetic investigation of communities by reconstruction of unobserved states
PTS: Phosphotransferase system
TCT: ThinPrep cytologic test
SD: Standard deviation
CHAO1: Chao1 richness estimator
ACE: Abundance-based coverage estimator
NSIL: Negative for squamous intraepithelial lesion
ASCUS: Atypical squamous cells of undetermined significance
ASC-H: Atypical squamous cells cannot exclude HSIL
TZ: Transformation zone
P16: Cyclin-dependent kinase inhibitor 2A
PH: Potential of hydrogen
HIV: Human immunodeficiency virus
PBS: Phosphate-buffered saline
DNA: Deoxyribonucleic acid
CTAB: Cetyltrimethylammonium bromide
PCR: Polymerase chain reaction
GC Buffer: Guanine-cytosine buffer
SD: Standard deviation
PCoA: Principal coordinate analysis
CST: Community state types
